# Key Collaborative Factors When Medicaid Accountable Care Organizations Work With Primary Care Clinics to Improve Colorectal Cancer Screening: Relationships, Data, and Quality Improvement Infrastructure

**DOI:** 10.5888/pcd16.180395

**Published:** 2019-08-15

**Authors:** Melinda M. Davis, Rose Gunn, Robyn Pham, Amy Wiser, Kristen Hassmiller Lich, Stephanie B. Wheeler, Gloria D. Coronado

**Affiliations:** 1 Oregon Rural Practice-based Research Network, Portland, Oregon; 2 Department of Family Medicine, Oregon Health and Science University, Portland, Oregon; 3 Department of Health Policy and Management, University of North Carolina at Chapel Hill, Chapel Hill, North Carolina; 4 Lineberger Comprehensive Cancer Center, University of North Carolina at Chapel Hill, Chapel Hill, North Carolina; 5 Center for Health Promotion and Disease Prevention, University of North Carolina at Chapel Hill, Chapel Hill, North Carolina; 6 Center for Health Research, Kaiser Permanente, Portland, Oregon

## Abstract

**Purpose:**

Accountable Care Organizations (ACOs) are implementing interventions to achieve triple-aim objectives of improved quality and experience of care while maintaining costs. Partnering across organizational boundaries is perceived as critical to ACO success.

**Methods:**

We conducted a comparative case study of 14 Medicaid ACOs in Oregon and their contracted primary care clinics using public performance data, key informant interviews, and consultation field notes. We focused on how ACOs work with clinics to improve colorectal cancer (CRC) screening — one incentivized performance metric.

**Results:**

ACOs implemented a broad spectrum of multi-component interventions designed to increase CRC screening. The most common interventions focused on reducing structural barriers (n = 12 ACOs), delivering provider assessment and feedback (n = 11), and providing patient reminders (n = 7). ACOs developed their processes and infrastructure for working with clinics over time. Facilitators of successful collaboration included a history of and commitment to collaboration (partnership); the ability to provide accurate data to prioritize action and monitor improvement (performance data), and supporting clinics’ reflective learning through facilitation, learning collaboratives; and support of ACO as well as clinic-based staffing (quality improvement infrastructure). Two unintended consequences of ACO–clinic partnership emerged: potential exclusion of smaller clinics and metric focus and fatigue.

**Conclusion:**

Our findings identified partnership, performance data, and quality improvement infrastructure as critical dimensions when Medicaid ACOs work with primary care to improve CRC screening. Findings may extend to other metric targets.

**Summary**

**What is already known on this topic?**

Partnering across organizational boundaries is critical to accountable care organization (ACO) success.

**What is added by this report?**

We explored how Oregon’s Medicaid ACOs are working with primary care clinics to improve the colorectal cancer (CRC) screening performance metric. We identified partnership, performance data, and quality improvement infrastructure as critical dimensions when ACOs and primary care clinics work to implement interventions to improve CRC screening. Unintended consequences included the potential exclusion of smaller clinics and metric focus and fatigue.

**What are the implications for public health practice?**

Practitioners looking to build cross-sector ACO–clinic partnerships to increase CRC screening or address other performance metrics should consider these 3 key collaborative factors and 2 unintended consequences.

## Introduction

Federal and state policies in the United States are increasingly moving away from pay for performance and toward reimbursement models that support the triple-aim objectives of improved quality and experience of care for populations while controlling costs (1). The Affordable Care Act, which was passed in March 2010, has many provisions that encourage achievement of the triple aim through expanded access to preventive care services, including encouraging cross-sector collaborations for care delivery through accountable care organizations (ACOs). ACOs are accountable for the quality and total costs of care for a defined population.

ACOs began in Medicare as a way to deliver high-quality, coordinated care; states have also expanded this model to Medicaid (2,3). Increased coordination and accountability in ACOs may lead to wiser spending and improved quality of care by delivering the right care to the right patient at the right time. In such cases, shared savings may be distributed across partner agencies (2). However, early research suggests there is considerable variation in partnership structures, decision making, and reimbursement models for ACOs (4,5). Moreover, the interventions that ACOs pursue and how they implement them may vary drastically and have implications for program effectiveness. Research suggests that ACO success will hinge on the ability of health care organizations to successfully partner across boundaries (6).

One quality indicator across many ACO and payer initiatives is colorectal cancer (CRC) screening (7,8). CRC is the second leading cause of cancer deaths in the United States, causing over 50,000 deaths annually (9). Guideline-concordant screening using endoscopic or fecal testing options can reduce CRC morbidity and mortality rates and is cost-effective (10,11). However, little research explores what interventions ACOs implement to increase CRC screening or how they work with primary care clinics.

Therefore, we sought to understand how ACOs work with primary care clinics to improve CRC screening. We focused on Oregon because of the opportunity to analyze 16 Medicaid ACOs (called coordinated care organizations or “CCOs”) to understand 1) which types of interventions CCOs are using to improve CRC screening rates and 2) how CCOs work with primary care clinics to implement the target interventions. Our study was designed to be hypothesis generating and to suggest promising practices to facilitate effective ACO–clinic partnerships to achieve performance benchmarks for CRC screening.

## Methods

In 2011 the Oregon legislature passed House Bill 3650, authorizing the formation of CCOs. By statute, CCOs are governed by a partnership between those taking financial risk, professionals in the local health system (eg, doctors, hospitals), and community members; no CCO directly owns primary care clinics (12). CRC screening has been a CCO quality incentive metric since program inception, with annual reporting initiated in 2013. 

We conducted a cross-case comparative study of CCOs in Oregon by using public performance data, transcripts from key informant interviews, and field notes from technical assistance consultations with CCO leadership. Our study was conducted in 2016, four years after CCO formation began. The institutional review board at Oregon Health and Science University approved this study (no. 11454).

### Data collection and participant sampling

First, we collected publicly reported data about CCO characteristics and CRC screening performance in early 2016; we added 2016 CRC screening rates when they became available in 2017. Second, 2 members of the study team (M.M.D., R.P.) conducted CRC technical assistance consultation meetings with CCO leadership and quality improvement teams during June and July of 2016. Finally, one member of the study team (M.M.D.) conducted key informant interviews with a purposive sample of stakeholders from CCOs, primary care clinics, and the state from February 2016 through August 2016. Interviews followed a semistructured guide designed to clarify our understanding of how CCOs worked with clinics to address the CRC incentive metric. Interviews lasted approximately 60 minutes (range, 31–118 min) and were audio recorded and professionally transcribed.

### Data management and analysis

Interview transcripts were checked for accuracy, and data were de-identified and analyzed using Atlas.ti version 7.0 (Atlas.ti Scientific Software). We found that existing conceptual frameworks and models did not account for the developmental nature of ACO and clinic partnerships over time (13). Therefore, we analyzed our data inductively to allow key themes to emerge naturally from the data.

We collected and analyzed data concurrently until saturation was reached (14). We used an iterative approach informed by Crabtree and Miller’s 5-stage immersion-crystallization analysis process (15). First, 2 authors (M.M.D., R.P.) reviewed transcripts and coded key segments of text with descriptive names (eg, partnership development, intervention targets) using a group process. Second, we reviewed data from a single CCO to understand how the organization was approaching CRC screening improvement and how they engaged primary care clinics and other stakeholders in this work. In a third cycle, 3 authors (M.M.D., R.P., R.G.) conducted a cross-case comparative analysis to identify patterns in CCO–clinic partnerships and associated performance on the CRC screening metric. We refined emerging themes with the larger study team and shared preliminary findings with OHA staff as a form of member checking (16). Use of reflexivity, multiple reviewers, data saturation, and an audit trail are associated with rigor in qualitative research methods (14,17).

## Results

In 2015 the 16 CCOs ranged in size from 11,347 to 228,263 Medicaid enrollees and had an average CRC screening rate of 46.4% (Table 1). Qualitative data were gathered from 14 CCOs (88% response rate). Thirty-eight informants representing 10 CCOs participated in technical assistance consultations; 26 stakeholders representing 12 CCOs participated in key informant interviews. Interview participants represented CCO leadership (n = 16), primary care clinics (n = 6), and the state (n = 4).

**Table 1:** Characteristics of Oregon’s Medicaid Accountable Care Organizations,^a^ Public Data 2015

Organization Name	Structure	Nonprofit Status	Number of Enrollees	Enrollee composition			
				% White	% Hispanic	% African American	% With a Disability
AllCare Health Plan	Corporation	No	48,790	71.7	10.5	0.9	6.5
Cascade Health Alliance	LLC	No	16,439	65.7	15.2	1.4	8.0
Columbia Pacific	LLC	Yes	24,975	72.7	9.6	0.7	6.3
Eastern Oregon	LLC	No	47,651	58.7	24.2	0.8	6.3
FamilyCare	Corporation	Yes	123,084	51.1	15.9	5.7	2.8
Health Share of Oregon	Corporation	Yes	228,263	49.9	18.0	7.8	8.5
Intercommunity Health Network	Corporation	Yes	54,679	69.4	10.7	0.8	7.8
Jackson Care Connect	LLC	Yes	29,157	64.5	15.8	0.9	6.1
PacificSource–Central Oregon	Corporation	Yes	51,973	70.6	12.2	0.6	5.5
PacificSource–Gorge	Corporation	Yes	12,833	52.3	33.3	0.6	4.8
PrimaryHealth of Josephine County	LLC	Yes	11,347	73.9	7.9	0.6	6.7
Trillium^b^	Corporation	No	90,564	70.1	9.8	1.8	8.3
Umpqua Health Alliance^b^	LLC	No	26,203	79.4	6.2	0.5	8.6
Western Oregon Advanced Health	LLC	No	20,048	77.3	7.4	0.6	9.9
Willamette Valley Community Health	LLC	No	98,112	51.5	28.5	1.4	6.6
Yamhill Community Care	Corporation	Yes	22,466	62.4	20.7	0.7	4.0

Abbreviation: LLC, limited liability corporation.

a Also known as coordinated care organizations (CCOs).

b Not included in subsequent analyses due to lack of qualitative data. Of the 16 CCOs, 14 participated in either the CCO technical assistance consultation or key informant interviews or both.

Participating CCOs were actively implementing multiple intervention strategies, including those to increase community demand, increase community access, and increase provider delivery of CRC (Table 2). The most common intervention strategies were reducing structural barriers (85.7%, n = 12), delivering provider assessment and feedback (78.6%, n = 11), and offering patient reminders (50.0%, n = 7). All 14 CCOs implemented intervention strategies with sufficient evidence of effectiveness according to the Community Guide (www.thecommunityguide.org); more than half (n = 8) were also implementing interventions with insufficient evidence.

**Table 2:** Interventions Being Implemented by Oregon CCOs, Reported in 2016 (N = 14)^a^

CCO ID^b^	Increase Community Demand					Increase Community Access^d^	Increase Provider Delivery			CRC Screening Rate		
	Patient Reminders^c^	Patient Incentives	Small Media^c^	Mass Media	One-on-One Education^c^	Reducing Structural Barriers^c^	Provider Assessment and Feedback^c^	Provider Reminder and Recall^c^	Provider Incentives	2014	2015	2016
A	X		X			X	X	X		53.3	51.7	52.8
B	X					X				47.4	48.8	52.6
C		X				X	X			47.0	47.8	48.5
E							X	X		46.7	47.3	51.1
F						X	X		X	48.4	49.9	55.0
G						X	X		X	46.7	49.4	49.9
H		X	X			X	X			54.0	43.8	51.8
I	X		X		X	X	X		X	35.3	36.0	40.9
J	X	X			X		X	X	X	29.7	38.7	43.1
K	X				X	X	X	X		31.6	46.6	47.9
M						X	X	X		53.5	49.0	50.6
N				X		X				51.8	49.1	54.5
O	X		X			X		X		52.1	47.7	47.4
P	X		X	X		X	X			40.5	44.3	53.5

Abbreviations: CCO, coordinated care organization; CRC, colorectal cancer; ID, identification.

a The interventions identified are provided by and defined from the Community Guide (www.communityguide.org).

b Qualitative data was not available for CCOs D or L.

c Intervention with sufficient evidence of effectiveness.

d Reducing patient out-of-pocket costs (an intervention with insufficient evidence of effectiveness) does not appear in the table because it did not emerge as an intervention being implemented by any CCO.

CCOs addressed 3 key areas when working with primary care clinics to improve CRC screening: 1) establishing relationships and building partnerships, 2) producing and sharing performance data, and 3) developing a process and infrastructure to support quality improvement (Figure). Illustrative quotes detailing these themes are in Table 3.

**Figure:**
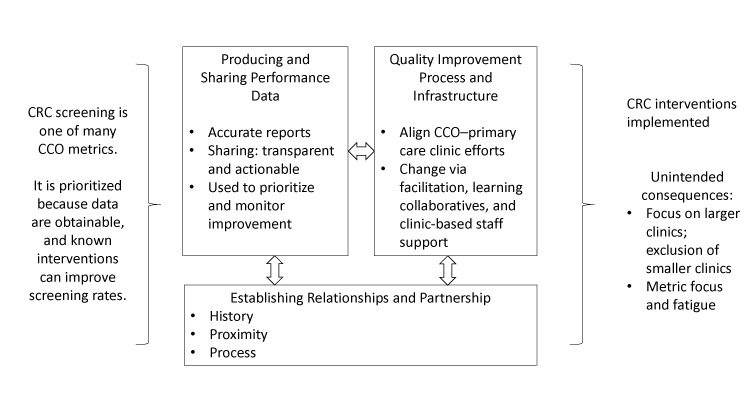
Three key collaborative factors when Medicaid accountable care organizations work with primary care clinics to achieve performance metrics for CRC screening. Abbreviations: CRC, colorectal cancer; CCO, coordinated care organization.

**Table 3:** Illustrative Quotes for Key Themes and Unintended Consequences

Theme	Illustrative Quotes
**Key factors in CCO–clinic collaborations**	
Establishing relationships and building partnerships	[CCO Name] is cited as a real pioneer in this work. . . . They have had such incredible community investment from the very beginning. It's not like they have to talk their partners into doing something or engaging in work around the metrics . . . because the partners were there from the beginning and were part of the founding governing board. (P12) We were really close with some clinics, and they trusted us. And some clinics, we didn’t have as close a relationship. So we had to figure that out in the strategy. [Now our relationships are] pretty close . . . partially because there’s a lot of need and they realize that we want to help. We don’t have some crazy ulterior motive. Our motive is the same as theirs. We want access for patients and quality care. (P7)
Producing and sharing performance data	We’ve gotten more sophisticated about [our process of sharing performance data]. . . . We identified somebody at the clinic that’s our contact. It may be administration or care management, it’s not necessarily going to be the primary care provider anymore. (P10) This CCO puts [performance data] in front of all of the providers on a regular basis. This is how you’re doing, this is how the clinic next to you is doing, this is how the clinic down the street is doing. I would have thought that would have been very risky, but . . . [it] has generated competition and it's generated transparency and it’s generated a spirit of collaboration because clinics can look at each other and say, “Boy, you’re doing great. Tell me what your secret is and let’s figure this out together, and will you help us? What did you do to get from here to here?” (P12)
Developing a process and infrastructure to support quality improvement	[We consider] each clinic and say, “For this clinic, what is it for them?” They’ve already got strong leadership, so maybe for them it’s that their data system makes it really difficult for them to track this metric. . . . We try to personalize our knowledge of each clinic to ensure that when we take something that seems straightforward, like they just need to improve the numerator hits for this process and it seems straightforward because you should just send out kits and they should get sent back but there’s always more beneath the surface. And typically what’s underneath it is some kind of system support that is not in place. (P4) Our first step is usually to educate the providers and their staff on what the quality measures are, how they are tracked, what kind of data are OHA looking for and what documentation do they need and the clinical record to back up that information . . . and then looking at what kind of clinical workflows or other strategies we can suggest to them or help them with that would improve the actual frequency in which services are occurring. (P16) It’s those kinds of hard stories that the clinics aren’t afraid to share [at the learning collaboratives] once we’ve developed trust . . . where they feel comfortable sharing their failures with each other, so you’re not [going] down the street reinventing the same crooked wheel. (P3)
**Unintended consequences**	
Engaging larger clinics, exclusion of smaller clinics	I feel for [these small clinics], because I think they're at a disadvantage in that larger clinics have built-in infrastructure of IT people, of performance improvement people, 3-tier leadership. . . . In some clinics, the office manager is the billing manager, is the front desk manager, is everything. I worry about those clinics and I wonder how they are doing. I don’t know if that falls on the CCO to provide that sort of infrastructure. Maybe it does. I just worry that they're being overlooked. (P22) We have really good reporting. . . . We have gap lists that we can produce by clinic, by provider, by measure. We know who’s got the most members for that measure, who’s contributing the most to the numerator and to the denominator so that we know where to target. Usually you would just go, “Oh, let’s let everybody know that we don’t, or everybody has to have them.” Well, now we go, “Okay, if we approach this one clinic, we can get everything we need to make the measure.” . . . We’re just being very strategic about that. (P10)
Metric focus and fatigue	For good or for bad, I think the metrics are really driving a lot of the effort now, and if there’s any bandwidth leftover after you’ve hit the metrics, then they focus on those things that don’t necessarily impact the check at the end of the year. . . . Somebody said just a couple of weeks ago, “I thought this would get easier. I thought it would calm down. I thought it would become more routine functioning, and it isn’t.” It is intense work, and it has been from the beginning. (P12) That’s probably the biggest thing that hit the clinics with new metrics, which is one more thing. “We just are barely getting this other thing working, and now you want us to do one more, you want us to do 2 more, and 3 more things,” and that’s the hard part. (P15) There's just too many [metrics], and the administrative burden of capturing the data for many of them . . . is too much. So it deters from true quality, and it deters from CCOs being able to focus on things that aren't quality metrics that could improve quality even more because quality isn’t just about quality metrics. (P7)

Abbreviations: CCO, coordinated care organization; IT, information technology; OHA, Oregon Health Authority; P, participant.

### Establishing relationships and building partnerships

 Relationships played an important role in shaping how CCOs interacted with primary care clinics in their service region and in their ability to make improvements. Relationship quality could facilitate as well as impede the selection and implementation of interventions to increase CRC screening.

Prior history between CCO leadership and primary care stakeholders, physical proximity of the CCO’s infrastructure, and joint leadership roles in the CCO and regional clinics shaped the tenor of these relationships. One stakeholder noted, “We really just try to build the bond and leverage our existing relationships. . . . We had an advantage to be able to walk into the clinics and have a pretty long history of trust” (Participant 18). In contrast, CCOs that built on less-developed partnerships, strained relationships, or those that lacked a physical presence in the community faced challenges in raising local awareness and building trust.

CCOs developed or built on their relationships and partnerships with primary care clinics over time in 4 key ways. First, they had primary care providers and clinic leadership serve on the CCO board or on various subcommittees. Second, they hired local staff to provide ongoing support and to facilitate change in the primary care clinics. Third, CCO staff spent time listening, building trust, and aligning CCO initiatives with health system–level and clinic-level priorities and needs. Finally, CCOs created or expanded permanent physical space to house their staff in the local communities served.

### Producing and sharing performance data

 Performance data provided a starting point to prioritize and direct improvement activities for the CCOs and their contracted primary care clinics. CCOs used CRC screening data to inform targeted clinic outreach; motivate improvement at the clinic, provider, and team levels; and monitor progress toward performance goals. A first step was to obtain and share accurate performance data with clinics. One CCO medical director commented, “I think everyone assumes they’re doing a good job, until we can present them with credible evidence otherwise” (Participant 14). Some CCOs withheld incentive metric resources in early years to “put a system together to provide data to our partners so that they could do that improvement work on accurate, reliable data” (Participant 25).

CCO leadership anticipated that routinely sharing performance data and gap lists for CRC screening would enable clinics to “scrub their schedules as people are coming in or be reaching out to patients [using a population management approach]” (Participant 16). Over time, CCOs learned to be more strategic in how they distributed the gap lists for CRC screening — in terms of who at the clinic received them and how the data were presented and/or accessed — and they created processes to increase data accuracy by enabling clinics to amend CCO claims data with historical screening recorders. Low-quality, inaccurate data were poorly received by clinic partners. One clinic member said, “We would get reams of paper, and about the fourth or fifth page in when three-quarters [of the patients] . . . weren't assigned to us we saw them as un-useful and put them aside” (Participant 8).

CCOs that had good standing relationships with clinics and the ability to generate metric data could also promote friendly, productive competition with transparent reporting of metric performance data, as illustrated in the following quote:

[Routine sharing of identified performance data] has generated competition, transparency, and a spirit of collaboration. Clinics can look at each other and say, “Boy, you're doing great. Tell me what your secret is and let's figure this out together, and will you help us? What did you do to get from here to here?” (Participant 12)

However, clinics varied in their ability to respond to performance data. Some clinics distributed performance data to panel managers who would then reach out to patients. Without dedicated staff to process or act on the CCO reports, the data languished at a clinic.

### Developing a process and infrastructure to support quality improvement

Relationships and data allowed CCOs to partner with clinics and health system leadership to focus on quality improvement initiatives at the clinic level. CCO-funded regionally based improvement staff focused on building relationships and supporting clinics as they worked to achieve the incentive metrics, including CRC screening. One CCO improvement facilitator described how the metrics were straightforward to understand, but the approach to achieve these metrics at each clinic required targeted support. Facilitators described asking, “What does it take for this clinic to implement this?” (Participant 4), then building a tailored improvement approach that attended to clinic needs (eg, addressing leadership, understanding and using data, improving team functioning). Improvement facilitators often began by educating providers on the quality metrics then helping clinics refine clinical workflows or implement strategies to improve service delivery frequency.

Some CCOs also led regional learning collaboratives and funded clinic-based quality improvement staff. Learning collaboratives allowed quality improvement leads and staff from regional clinics to gather and share best practices, troubleshoot workflows, and plan their own initiatives. Clinic-based quality improvement staff helped lead clinic change or were panel managers who performed key tasks to support improvement efforts for CRC screening and other incentive metrics.

### Promising practices

Despite heterogeneity in interventions implemented across CCOs, certain patterns stood out as promising in relation to CCO–clinic partnerships to improve CRC screening. Stakeholders noted how certain CCOs leveraged their relationships with partner clinics or funded staff to help implement changes in care delivery needed to achieve CRC screening metric benchmarks. The ability to provide accurate data to prioritize action and improvement monitoring was also critical. However, clinics also needed a process for acting on this information. Although some clinics had robust quality improvement infrastructure, others needed resources and training to be able to review data, select interventions, and implement changes. In contrast, some CCOs with lower levels of clinic engagement and data reporting or sharing capacity implemented CRC initiatives that circumvented clinics (eg, offering fecal tests for CRC screening directly to Medicaid enrollees). Although CCO-led interventions could increase CRC screening rates, informants indicated that this approach contributed to over-screening by duplicating clinic-level workflows, raised concerns about legal ramifications in relation to patient follow-up on abnormal results, and reduced the willingness of clinics and health systems to collaborate.

### Unintended consequences

Collaboration between CCOs and clinics suggested 2 emerging and unintended consequences: 1) prioritizing larger clinics and excluding smaller clinics and 2) metric focus and fatigue. The ability to generate high-quality data and the need to build relationships and quality improvement infrastructure led many CCOs to focus their attention and resources on larger clinics. Stakeholders expressed concern that some of the smaller clinics — which may have more limited quality improvement capacity to begin with and are often found in rural areas where screening disparities exist — were not given data reports from the CCO or support with improvement. One stakeholder commented, “Sadly, I think if you look at the large clinics that are doing well . . . we consider[ed] that a win and we move[d] on. I would hate for someone to not be screened [for CRC] just because of the clinic they chose” (participant 22).

A second unintended consequence was a focus on the CCO metrics to the exclusion of other factors associated with quality of care and feelings of metric fatigue. Stakeholders commented on the number of metrics that clinics are responsible for, the burden of capturing and reporting data, and the pressure for continual improvement. “People are just exhausted. They come to the end of a metric year and . . . it's like fighting with every ounce of energy you have to make sure that you’ve got enough people under your belt to hit a particular metric” (participant 12).

## Discussion

Our study explored how Medicaid ACOs (CCOs in Oregon) work with primary care clinics to improve CRC screening. CCOs addressed 3 key collaborative factors: establishing relationships and building partnerships, producing and sharing performance data, and developing quality improvement processes and infrastructure. All CCOs were implementing multi-component interventions, some with sufficient evidence and others with insufficient evidence of effectiveness. Access to and knowledge of the performance metrics and an expectation that clinics would take action to increase CRC screening improvement was necessary but not sufficient. Robust relationships, high-quality actionable data, and helping clinics fund and figure out how to make improvements are promising practices associated with enhanced CCO–clinic collaboration to increase CRC screening.

Two unintended consequences emerged in our exploration of CCO–clinic partnerships that warrant additional attention. First, neglect or exclusion of smaller clinics may increase CRC screening disparities, and smaller clinics may experience more barriers to implementing change (18–20). Including smaller clinics is critical in supporting improved care, given that 78% of patients in the United States still receive care in clinics with 10 or fewer physicians (21). Second, metric focus and fatigue suggests the need to attend proactively to provider and staff burnout, to support team-based care models, and to stay cognizant of what “gets missed” as ACOs and CCOs focus on quality metrics at the potential expense of quality (22).

Our study contributes to a growing body of literature on effective practices for ACOs and to the broader literature on cross-sector partnerships and multi-level interventions using CRC as a case study. Findings encourage use of participatory approaches that attend to local context and needs (23,24) and support improvement as a dynamic process within a complex system using a “best processes” orientation (25).

Two areas warrant additional consideration. First, our findings highlight the opportunities and challenges of building cross-sector partnerships to implement interventions that increase CRC screening. Stakeholders described the importance of building trusting relationships and basic infrastructure as part of efforts to implement evidence-based interventions in routine care. Although ACOs may want to focus on specific interventions first, building basic improvement capacity can lay the foundation for successful implementation later. Second, although selecting an evidence-based intervention is a key component of improvement practice, determining how to support implementation is a critical determinant of intervention success. Facilitation — or providing support to aid implementation — is increasingly recognized as a critical factor of implementation success (26,27). Facilitators may engage key partners to implement needed change, to create a safe space for data sharing and reflection on improvement targets, and to optimize intervention delivery and understanding over time (28,29). Finally, our study findings suggest that in certain cases ACOs may also need to provide internal staffing support to enable clinics to implement interventions to achieve performance benchmarks. Even if well-intentioned, providing technical support without considering how to resource or to reward clinics and staff for making change may be poorly received and lack anticipated impact (30).

Our study has limitations. First, our data were cross-sectional. Although stakeholders described how CCOs were evolving their strategies over time, we were not able to evaluate these changes in detail or to definitively identify successful and unsuccessful intervention or implementation strategies. Future studies would benefit from assessing changes in CCO approaches over time, and their association with performance metrics. Also, we focused on how CCOs worked with primary care clinics on one metric, CRC screening. It is possible that different metrics may require other strategies to address. Regardless, our findings are likely generalizable to other preventive screenings.

Partnerships are perceived as critical to ACO success. We found that Oregon Medicaid ACOs engaged with primary care clinics to improve CRC screening by implementing multi-component interventions (eg, reducing structural barriers, delivering provider assessment and feedback, providing patient reminders). Facilitators of successful collaboration included a history of and a commitment to collaboration, the ability to provide accurate data to prioritize action and monitor improvement, and supporting clinics’ reflective learning through facilitation, learning collaboratives, and support of clinic-based staff. Perceived exclusion of smaller clinics and metric focus and fatigue emerged as unintended consequences of these improvement efforts and warrant additional attention. ACO–clinic partnerships must go beyond simply sharing what is needed for improvement to helping clinics figure out how to make improvements, which may include resourcing external and internal infrastructure. Our findings can inform ACOs how to effectively partner with primary care clinics to improve CRC screening and may extend to other performance metrics.
